# Implante Percutâneo de Válvula Aórtica Auxiliada pela Oxigenação por Membrana Extracorpórea para Tratamento de Estenose Aórtica com Choque Cardiogênico

**DOI:** 10.36660/abc.20201358

**Published:** 2021-07-14

**Authors:** Gangping Huang, Huaidong Chen, Weimin Zhang, Fan He

**Affiliations:** 1 Universidade de Zhejiang Hospital Sir Run Run Shaw Departamento de Cirurgia Cardíaca Zhejiang China Departamento de Cirurgia Cardíaca, Hospital Sir Run Run Shaw, Escola de Medicina, Universidade de Zhejiang, Zhejiang - China

**Keywords:** Oxigenação por Membrana Extracorpórea, Estenose da Valva Aórtica, Choque cardiogênico

## Introdução

A estenose aórtica é uma doença valvular cardíaca comum, geralmente causada por uma doença degenerativa da válvula aórtica nos idosos.^1^ A estenose aórtica obstrui o fluxo para a frente do ventrículo esquerdo até a aorta, levando a um diferencial de pressão entre o ventrículo esquerdo e a aorta e o aumento da pressão do ventrículo esquerdo secundário à hipertrofia do ventrículo esquerdo. À medida que a doença avança, ela leva à disfunção diastólica e sistólica do ventrículo esquerdo e à isquemia miocárdica devido à diminuição do fluxo sanguíneo coronário. Entretanto, o choque cardiogênico secundário à estenose aórtica é uma das complicações mais sérias e tem um alto índice de mortalidade devido a seu efeito terapêutico limitado.^2^ Desde seu início em 2002, o implante percutâneo da válvula aórtica (TAVI) se tornou o tratamento de escolha para pacientes idosos com estenose aórtica grave e alto risco cirúrgico devido a suas vantagens de ser minimamente invasivo, circulação não extracorpórea, e boa eficácia de meio e longo prazo.^3^^-^^5^ Com o desenvolvimento da tecnologia dos dispositivos e sistemas de transmissão de baixa resistência, o TAVI demonstrou recentemente ser tão eficiente do que a cirurgia tradicional, mesmo em pacientes de risco médio.^6^ Entretanto, para pacientes com histórico médico longo, ele diminuiu significativamente a fração de ejeção cardíaca (FE), choque cardiogênico, doença da válvula aórtica descompensada e complicações graves, tais como a instabilidade hemodinâmica intraoperatória e arritmia maligna, ainda existem durante a cirurgia com TAVI, aumentando significativamente o risco de TAVI. Neste estudo, relata-se um caso de estenose aórtica grave complicada por choque cardiogênico que foi tratado com sucesso com TAVI auxiliado pela oxigenação por membrana extracorpórea.

## Relato de Caso

**Figura 1:**
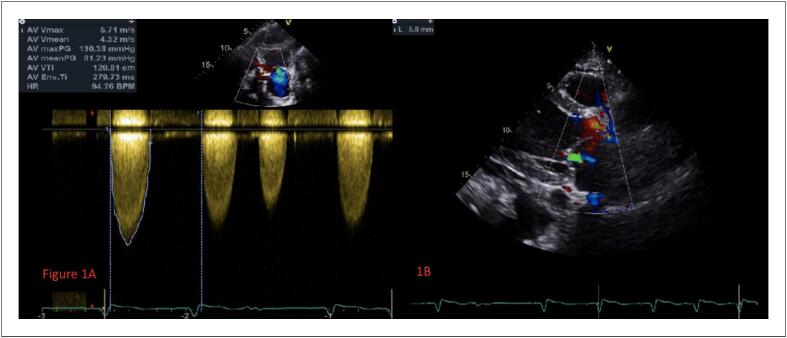
A e B) O ecocardiograma pré-operatório indicado indicou que o gradiente de pressão e a velocidade da válvula aórtica aumentaram, a abertura da válvula aórtica se calcificou, e ocorreu estenose aórtica grave.

**Figura 2:**
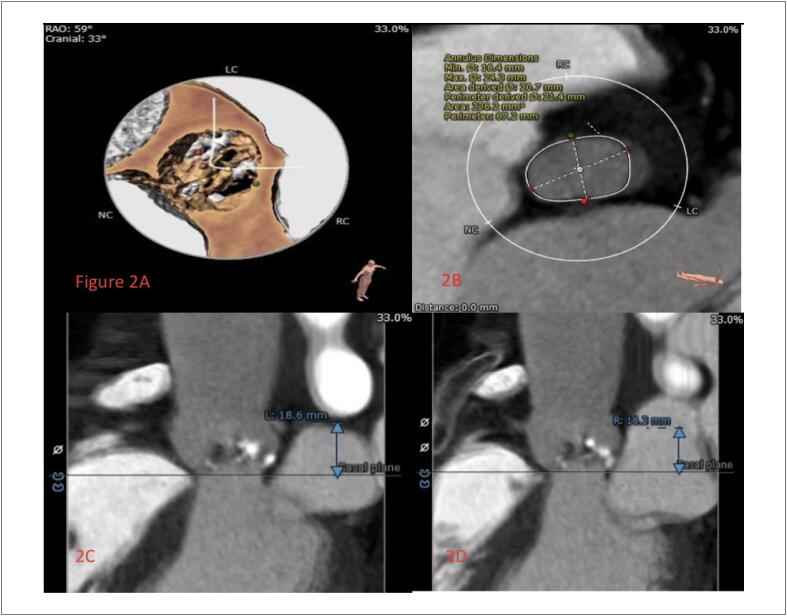
Reconstrução tridimensional da válvula aórtica (2A), diâmetro anular aórtico (2B), altura do óstio coronário esquerdo (2C), e óstio coronário direito (2D).

**Figura 3:**
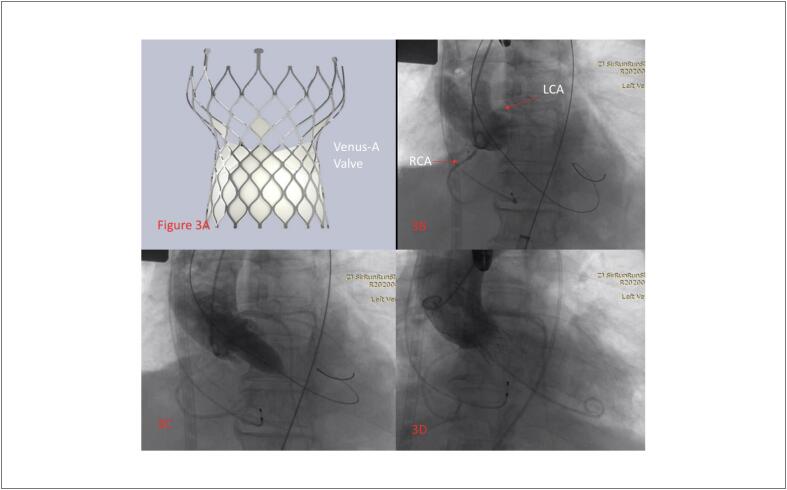
A) A válvula utilizada era da China; B) Aortografia antes da dilatação do balão indicou que as artérias coronárias estavam bem desenvolvidas, e que havia estenose grave da válvula aórtica; C) Aortografia com o balão totalmente inflado mostrando a patência das artérias coronárias esquerda e direita; D) Aortografia final após a instalação da válvula e dilatação demonstrou um bom posicionamento da válvula e uma pequena quantidade de vazamento perivalvular.

Uma paciente de 64 anos, do sexo feminino, foi hospitalizada devido a “pressão no peito e fadiga por mais de 2 meses e agravamento por 3 dias”. Ela tinha história prévia de colecistectomia por 10 anos. O exame físico na admissão indicou uma temperatura de 36,8 °C, 18 respirações/minuto, pulso de 46 batimentos/minuto, e pressão arterial de 136/92 mmHg. A paciente tinha ortopneia. Os sons pulmonares de ambos os lados eram grossos, e podia ser ouvido um ronco úmido. O sopro sistólico podia ser ouvido na auscultação da válvula aórtica e havia um ligeiro linfedema em ambas as pernas. Exames laboratoriais mostraram peptídeo natriurético pró-cerebral N-terminal (NT-proBNP)>25000 pg/mL, Troponina I (TnI) 0,12 μg/L. Os índices de função renal e hepática também aumentaram significativamente. O ecocardiograma sugeriu estenose aórtica grave com insuficiência leve, o gradiente de pressão sistólica máximo era de 130mmHg e o ventrículo esquerdo foi significativamente aumentado (LVIDd:58,3mm) com disfunção diastólica e sistólica. A FE foi medida em 23,5% pelo método biplanar (Figura 1). O TC do tórax demonstrou exsudato pleural duplo, edema intersticial pulmonar, efusão encapsulada de ambos os pulmões, com insuficiência pulmonar. O ECG dinâmico de 24 horas sugere o ritmo sinusal com batimentos prematuros atriais e ventriculares frequentes. Os resultados da aferição da pressão arterial ambulatorial demonstrou que a pressão arterial foi de 96/64 mmHg durante o dia, 98/65 mmHg durante o dia, e 93/62 mmHg à noite. A avaliação do TAVI por imagens dos pacientes, e os resultados demonstraram estenose aórtica típica com malformação bicúspide (tipo 0) e calcificação moderada (Figura 2). Os diagnósticos foram estenose aórtica e choque cardiogênico com uma função cardíaca de classificação NYHA IV. O paciente recebeu cardiotônicos, diuréticos, ventilação não invasiva adjuvante, mas a insuficiência cardíaca e os sintomas respiratórios não melhoraram. Considerando que o paciente tinha alto risco de choque cardiogênico e insuficiência cardíaca causada pela estenose aórtica, não houve condições de realização de uma cirurgia aberta. O risco da cirurgia TAVI também era muito alto. Então, depois de uma consulta multidisciplinar, foi proposto um plano de tratamento com cirurgia TAVI auxiliada por ECMO. A intubação traqueal foi inserida sob anestesia geral. O cateter guia 6F estava intravesical à veia jugular direita, e, em seguida, o marca-passo temporário foi inserido no ventrículo direito pelo cateter guia. A artéria femoral direita foi puncionada com uma agulha de microperfuração 4F, o cateter, de 6F a 11F, foi utilizado para expandir, e 2 dispositivos de fechamento Perclose Proglide (Abbott Vascular, Minneapolis, MN, EUA) foram inseridos para aplicação em standby, e, em seguida, o tubo 18F foi inserido. A oxigenação por membrana extracorpórea venoarterial (VA-ECMO) foi realizada inserindo-se uma cânula arterial 16F e uma cânula venosa 22F na artéria femoral esquerda e na veia femoral, respectivamente. O fluxo assistido por circulação era de 2,7 L/min, e a pressão arterial foi mantida em aproximadamente 120 mmHg. Durante a operação, é necessário garantir que a posição das cânulas arterial e venosa está em boa condição para evitar puxões, dobras, deslocamento e prolapso. Também é importante observar a cor do sangue e a tensão do lúmen, se há instabilidade no lúmen, se há coágulos sanguíneos, e se a hemodinâmica está estável durante o período de circulação assistida. Um cateter foi colocado no ventrículo esquerdo via o cateter guia da artéria radial esquerda e apresentou pressão de VE de 167/25 mmHg e pressão arterial aórtica de 100/77 mmHg. O fio guia foi enviado ao ventrículo esquerdo pelo cateter guia 18F e, em seguida, colocado no balão Numed 18 mm e expandido após definir o ritmo cardíaco temporário em 180 batimentos/min. Baseado nos dados medidos da reconstrução de TC, foi selecionada uma válvula Venus A-Valve de 23 mm (Venus MedTech, Hangzhou, China). A válvula foi liberada em um posicionamento preciso e ritmo cardíaco temporário de 160 batimentos/min. Os resultados indicaram que a forma e a posição da válvula eram boas, e a angiografia indicou uma pequena quantidade de vazamento perivascular (Figura 3).

**Figura 4:**
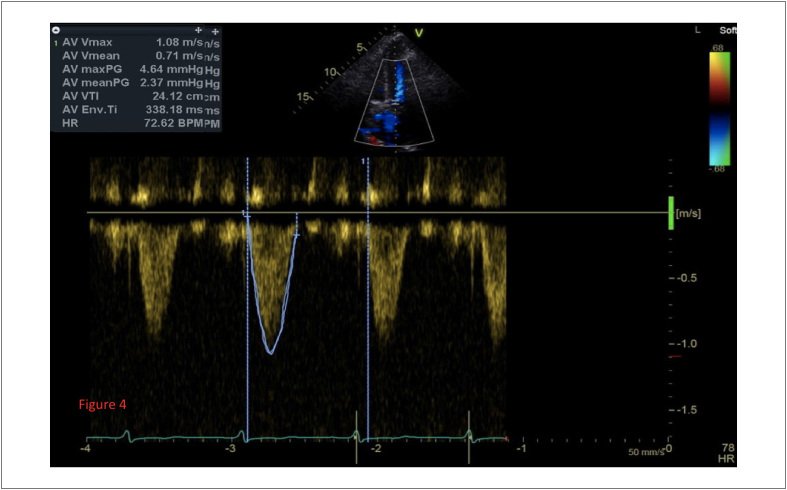
O ecocardiograma pós-operatório indicado indicou que o gradiente de pressão e a velocidade da válvula aórtica melhoraram significativamente.

Após o procedimento, o paciente, confiante no suporte total do ECMO e de fármacos vasoativos para manter a hemodinâmica, foi transferido para a unidade de terapia intensiva (UTI). A hemodinâmica do paciente estava estável e o ECMO foi retirado 20 horas após a cirurgia. Devido à atelectasia pré-operatória e ao edema pulmonar, a intubação traqueal foi extraída três dias após a operação. Os sintomas e sinais pós-operatórios do paciente melhoraram significativamente, os índices de NT-proBNP, TnI, e de função hepática e renal diminuíram significativamente. O ecocardiograma pós-operatório indicou uma função valvular normal acompanhada de uma pequena quantidade de vazamento perivalvular. A velocidade do orifício e o gradiente de pressão foram reduzidos significativamente em comparação aos anteriores à cirurgia, e o FE aumentou para 66% (Figura 4). Devido às condições graves do paciente antes da cirurgia, acamamento pós-operatório longo, desnutrição e influência de drogas, ocorreram sintomas pós-operatórios, tais como transtorno de consciência, infecção pulmonar, e trombose venosa intermuscular bilateral das extremidades. Pela melhoria do equilíbrio do ambiente interno, nutrição, exercícios funcionais anti-infecção e de reabilitação pós-operatório, o paciente finalmente recebeu a alta com sucesso.

## Discussão

TAVI é uma nova tecnologia para tratamento de doença aórtica. Depois de mais de uma década de desenvolvimento, o TAVI surgiu como uma opção de tratamento atraente e menos invasiva para a estenose aórtica grave, e é superior ao tratamento farmacológico para pacientes que não podem ser submetidos à cirurgia convencional.^7^^,^^8^ Com avanços modernos em equipamentos, o TAVI demonstrou ser tão eficiente quanto a cirurgia tradicional em pacientes com risco intermediário.^6^ Entretanto, na prática clínica, alguns pacientes foram associados ao FE baixo, ventrículo esquerdo reduzido, choque cardiogênico, e outros sintomas antes da cirurgia, que, sem dúvidas, aumentaram o risco da cirurgia TAVI. Encontrar um tratamento seguro e eficiente para esses pacientes com risco muito alto é sempre um desafio para os cardiologistas. O ECMO é um dispositivo de suporte circulatório mecânico que tem sido usado para auxiliar o tratamento adjuvante do choque cardiogênico e ressuscitação cardiopulmonar causados por vários motivos nos últimos anos.^9^ Para pacientes com instabilidade hemodinâmica, o ECMO pode garantir fluxo sanguíneo e suprimento de oxigênio estáveis e, portanto, tratar a insuficiência cardíaca reversível com eficiência.^10^ Entretanto, para pacientes com estenose aórtica grave com choque cardiogênico, a experiência do ECMO no TAVI é limitada. Um estudo observacional^11^ examinou os resultados do TAVI transapical em pacientes com choque cardiogênico e detectou que a presença do choque cardiogênico aumentava significativamente a mortalidade 30 dias após o TAVI (19% choque cardiogênico vs. 5% sem choque cardiogênico; p = 0,02). Entretanto, o índice de mortalidade do TAVI no grupo com choque cardiogênico ainda era mais baixo do após a substituição de válvula aórtica convencional (19% vs. 26%), sugerindo que o TAVI pode ser uma opção de tratamento viável para choque cardiogênico. O paciente deste estudo apresentou sintomas de choque cardiogênico grave após a admissão, tais como hipotensão e ortopneia, e tinha um alto risco, com classificação STS de 30,06. A cirurgia de troca de válvula aórtica tradicional tem um risco muito alto. Entretanto, a cirurgia TAVI nesse momento também aumente, sem dúvidas, o risco de instabilidade hemodinâmica intraoperatória, arritmia maligna e até mesmo morte súbita. Além disso, tratamentos conservadores, tais como cardiotônicos, diuréticos, e ventilação mecânica não invasiva não conseguiram melhorar os sintomas do paciente, podendo levar à morte. Portanto, se ocorrer o colapso circulatório, ou o paciente parecer ser intolerante ao TAVI, não se deve hesitar em usar o ECMO. De acordo com nossa experiência prática, o uso intraoperatório do ECMO garante a estabilização da hemodinâmica, permite, a expansão repetida da válvula aórtica doente sem arritmias malignas, tais como taquicardia supraventricular e fibrilação ventricular, reduz significativamente o risco do procedimento do TAVI, reduz significativamente a irritabilidade cardíaca intraoperatória possível em etapas fundamentais da cirurgia, tais como a expansão do balão na válvula aórtica e a liberação da válvula interveniente, e garante efetivamente a segurança da cirurgia em pacientes de alto risco. Ao mesmo tempo, para pacientes com estenose aórtica grave, a pressão arterial foi melhorada em níveis diferentes após a dilatação do balão assistida por ECMO, evitando o risco de colapso circulatório causado pela dilatação do balão em um estado não protegido desses pacientes, de forma que os pacientes possam se beneficiar ao máximo.

Entretanto, a incidência de complicações associadas ao ECMO (tais como isquemia de membros inferiores, acidente vascular cerebral, lesão vascular, lesão renal aguda, sangramento, e infecção) é tão alta que é essencial, para o uso eficiente e racional do ECMO por uma equipe de saúde experiente.^12^ Por outro lado, após a retirada bem-sucedida do ECMO, deve-se prestar mais atenção ao controle pós-operatório para melhorar os resultados pós-operatórios, tais como o uso de medicamento para insuficiência cardíaca, intervenção abrangente em comorbidades, reabilitação cardíaca prolongada, e acompanhamento ambulatorial próximo. O paciente deste estudo apresentou transtorno de consciência pós-operatório, e o TC não revelou sinais de infarto cerebral ou hemorragia cerebral, que poderiam ser causados pelo uso excessivo de sedativos. Assim, tomamos a decisão de interromper o uso de sedativos e administramos terapia neurotrófica. A alta antecipada da unidade de terapia intensiva pode reduzir a incidência de infecção cruzada e infecção iatrogênica após a operação. Além disso, exercícios de reabilitação pós-operatória, tais como, sair da cama, vibração pulmonar e drenagem de secreção também podem evitar a infecção pulmonar e a trombose venosa de membros inferiores.

Concluindo, esta experiência pode oferecer uma solução para esses pacientes. Entretanto, ainda são necessários estudos conduzidos com grandes amostras para se encontrar o melhor tratamento.

## Conclusão

O TAVI assistido por ECMO pode ser uma escolha razoável para pacientes com estenose aórtica grave pré-operatório complicada por FE baixo, insuficiência cardíaca, ou até mesmo choque cardiogênico. Por enquanto, um controle pós-operatório razoável pode evitar efetivamente complicações relacionadas ao ECMO e melhorar o prognóstico dos pacientes.
