# Supplementary material for: Implante Percutâneo de Válvula Aórtica Auxiliada pela Oxigenação por Membrana Extracorpórea para Tratamento de Estenose Aórtica com Choque Cardiogênico
Source: Arq Bras Cardiol. 2021 Jul 14;117(1 Supl 1):33–7. [Article in Portuguese] doi: 10.36660/abc.20201358 (PMC8291898; doi:10.36660/abc.20201358)
Supplement: Translation

# Translation: Transcatheter Aortic Valve Implantation Assisted by Extracorporeal Membrane Oxygenation for the Treatment of Aortic Stenosis with Cardiogenic Shock

**Authors:** Gangping Huang, Huaidong Chen, Weimin Zhang, Fan He

**Affiliations:** 1 Zhejiang China Department of Cardiac Surgery, Sir Run Run Shaw Hospital, School of Medicine, Zhejiang University, Zhejiang - China

**Keywords:** Extracorporeal Membrane Oxygenation, Aortic Valve Stenosis, Cardiogenic Shock

## Introduction

Aortic stenosis is a common heart valve disease, mostly caused by degenerative aortic valve disease in the elderly.^1^ Aortic stenosis obstructs the forward flow of the left ventricle to the aorta, leading to a pressure differential between the left ventricle and the aorta and increased left ventricular pressure secondary to left ventricular hypertrophy. As the disease progresses, it leads to left ventricular systolic and diastolic dysfunction and myocardial ischemia due to decreased coronary blood flow. However, cardiogenic shock secondary to aortic stenosis is one of the most serious complications and has a high mortality rate due to its limited therapeutic effect.^2^ Since its inception in 2002, transcatheter aortic valve implantation (TAVI) has become the first choice of treatment for elderly patients with severe aortic stenosis and high surgical risk due to its advantages of minimally invasive, non-extracorporeal circulation, and good medium-long term efficacy.^3^^-^^5^ With the development of device technology and low-resistance transmission systems, TAVI has recently been shown to be no less effective than traditional surgery, even in medium-risk patients.^6^ However, for patients with a long medical history, significantly reduced cardiac ejection fraction (EF), cardiogenic shock, decompensated aortic valve disease, and severe complications such as intraoperative hemodynamic breakdown and malignant arrhythmia, still exist during TAVI surgery, greatly increasing the risk of TAVI. The present study reports a case of severe aortic stenosis complicated by cardiogenic shock that was successfully treated with TAVI assisted by extracorporeal membrane oxygenation.

## Case Report

A 64-year-old female patient was hospitalized due to “repeated chest tightness and fatigue for more than 2 months and aggravation for 3 days”. She had a previous history of cholecystectomy for 10 years. The physical examination upon admission indicated a temperature of 36.8°C, 18 breaths/min, a pulse of 46 beats/min, and blood pressure 136/92 mmHg. The patient had an orthopnoea. The breathing sounds of both lungs were coarse, and a moist rhonchus could be heard. Systolic blowing murmur could be heard in the aortic valve auscultation, and there was slight pitting edema over both legs. Laboratory examination showed N-terminal pro-brain natriuretic peptide (NT-proBNP)>25000 pg/mL, Troponin I(TnI) 0.12 μg/L. The indexes of liver and kidney function also increased significantly. Echocardiography suggested severe aortic stenosis with mild insufficiency, the maximum systolic pressure gradient was 130mmHg, and the left ventricle was significantly enlarged (LVIDd:58.3mm) with systolic diastolic dysfunction. The EF was measured as 23.5% by biplane method (Figure 1). Chest CT showed double pneumonia exudate, pulmonary interstitial edema, encapsulated effusion of both lungs with pulmonary insufficiency. A 24h dynamic ECG suggested a sinus rhythm with frequent atrial and ventricular premature beats. The results of ambutral blood pressure examination showed that the blood pressure was 96/64mmhg throughout the day, 98/65mmhg during the day, and 93/62mmhg at night. TAVI imaging evaluation of the patients was conducted, and the results showed typical aortic stenosis with bicuspid malformation (type 0) and moderate calcification (Figure 2). The diagnoses were aortic stenosis and cardiogenic shock with a heart function classification of NYHA IV. The patient was given cardiotonics, diuretics, non-invasive ventilator adjuvant treatment, but the heart failure and respiratory symptoms did not improve. Considering that the patient was at high risk of cardiogenic shock and heart failure caused by aortic stenosis, there were no conditions for routine open-heart surgery, and the risk of TAVI surgery was also very high. Thus, after a multidisciplinary consultation, we proposed the treatment plan for ECMO-assisted TAVI surgery. Tracheal intubation was inserted under general anesthesia. The 6F sheath tube was indwelling in the right jugular vein, and then the temporary pacemaker was inserted into the right ventricle through the sheath tube. The right femoral artery was punctured with a 4F micropuncture needle, and the sheath of 6F and 11F was used to expand, and 2 Perclose Proglide closure devices (Abbott Vascular, Minneapolis, MN) were inserted for standby application, and then the 18F sheath tube was inserted. Then venoarterial extracorporeal membrane oxygenation (VA-ECMO) was performed by inserting 16F arterial cannula and 22F venous cannula into the left femoral artery and femoral vein respectively. The circulation assisted flow was 2.7L/min, and the blood pressure was maintained at about 120mmHg. During the operation, it is necessary to ensure that the position of the artery and vein cannula is in good condition to prevent pulling, bending, displacement, and prolapse. It is also important to observe the blood color and tension of the lumen, whether the lumen is quivering, whether there is blood clot, and whether the hemodynamics is stable during the period of circulation assistance. A left ventricular catheter was placed via the left radial artery sheath and showed an LV pressure of 167/25mmHg and aortic blood pressure of 100/77 mmHg. The guide wire was sent into the left ventricle through the 18F sheath tube and then put into the 18mm Numed balloon and expanded after setting the temporary pacing rate to 180 beats/min. Based on measured data of the CT reconstruction, we selected the 23mm Venus A-Valve (Venus MedTech, Hangzhou, China). The valve was released under precise positioning and temporary pacing rate of 160 beats/min. The results indicated that the valve shape and position were good, and the angiography indicated a small amount of perivalvular leakage (Figure 3).

**Figure 1:** A and B) Preoperative echocardiography indicated that aortic valve velocity and pressure gradient increased, aortic valve lobe calcified, and severe aortic stenosis occurred.

**Figure 2:** Three-dimensional reconstruction of the aortic valve (2A), the aortic annular diameter (2B), height of the left coronary ostium (2C), and the right coronary ostium (2D).

**Figure 3:** A) The valve used was from China; B) Aortography before the balloon dilatation indicated that the coronary arteries were well developed and a severe stenosis of the aortic valve; C) Aortography with the balloon fully inflated showing the patency of both the left and right coronary arteries; D) The final aortography after valve deployment and dilation showed a good position of the valve and a small amount of perivalvular leakage.

After the procedure, the patient, reliant on full ECMO support and vasoactive drugs to maintain hemodynamics, was transferred to the intensive care unit (CCU). The hemodynamics of the patient was stable and ECMO was removed 20 hours after surgery. Due to preoperative atelectasis and pulmonary edema, tracheal intubation was extracted three days after the operation. Postoperative symptoms and signs of the patient were significantly improved, NT-proBNP, TnI and liver and kidney function indexes were significantly decreased. The postoperative echocardiography indicated a normal valve function accompanied by a small amount of perivalvular leakage. The valve orifice velocity and pressure gradient were significantly reduced compared with those before surgery and the EF increased to 66% (Figure 4). Due to the patient's severe condition before surgery, long postoperative bed time, malnutrition, and the influence of drugs, the postoperative symptoms such as consciousness disorder, pulmonary infection and bilateral intermuscular vein thrombosis of the lower extremities occurred. Through the improvement of internal environment balance, nutritional nerve, anti-infection, and rehabilitation function exercise after surgery, the patient was finally discharged successfully.

**Figure 4:** Postoperative echocardiography indicated that aortic valve velocity and pressure gradient improved significantly.

## Discussion

TAVI is a new technology for the treatment of aortic disease. After more than a decade of development, TAVI has emerged as an attractive, less invasive treatment option for severe aortic stenosis, and is superior to drug therapy for patients who cannot undergo conventional surgery.^7^^,^^8^ With modern advances in equipment, TAVI has proven to be no less effective than traditional surgery in patients with intermediate risk.^6^ However, in clinical practice, some patients were associated with low EF, small left ventricle, cardiogenic shock and other symptoms before surgery, which undoubtedly increased the risk of TAVI surgery. Finding a safe and effective treatment for these very high risk patients is always a challenge for cardiologists. ECMO is a mechanical circulatory support device, which has been used in the rescue adjuvant therapy of cardiogenic shock and cardiopulmonary resuscitation due to various reasons in recent years.^9^ For patients with hemodynamic instability, ECMO can provide stable blood flow and oxygen supply, thus effectively treating reversible heart failure.^10^ However, for high-risk aortic stenosis patients with cardiogenic shock, the experience of ECMO in TAVI is limited. An observational study^11^ examined the results of transapical TAVI in patients with cardiogenic shock, and found that the presence of cardiogenic shock significantly increased the mortality at 30 days after TAVI (19% cardiogenic shock vs. 5% non-cardiogenic shock; p = 0.02). However, the mortality rate of TAVI in the cardiogenic shock group was still lower than that after emergency routine aortic valve replacement (19% vs 26%), suggesting that TAVI may be a viable treatment option for cardiogenic shock. Our patient presented severe cardiogenic shock symptoms after admission, such as hypotension and orthopnoea, and was at high risk, with an STS score of 30.06. Traditional SVAR surgery carries a very high risk. However, TAVI surgery at this time also undoubtedly increases the risk of intraoperative hemodynamic instability, malignant arrhythmia, and even sudden death. In addition, conservative treatments, such as cardiotonics, diuretics, and non-invasive ventilator assisted ventilation failed to improve the patient's symptoms, the final result may also be death. Therefore if circulatory collapse occurs or the patient seems to be intolerable of TAVI, we should not hesitate to use ECMO. According to our practical experience, the intraoperative use of ECMO effectively guarantees stable hemodynamics, enables the repeated expansion of a diseased aortic valve with no malignant arrhythmias, such as supraventricular tachycardia and ventricular fibrillation appeared, greatly reduces the risk of TAVI procedures, significantly reduces possible intraoperative heart irritability in key surgical stages, such as balloon expansion of the aortic valve and the release of the intervening valve, and effectively guarantees the surgical safety of high-risk patients. At the same time, for patients with severe aortic stenosis, blood pressure was improved to different degrees after balloon dilation assisted by ECMO, avoiding the risk of circulatory collapse caused by balloon dilation in the unprotected state of such patients, so that the patients could benefit to the maximum extent.

However, the incidence of ECMO-related complications (such as lower limb ischemia, stroke, vascular injury, acute kidney injury, bleeding, and infection) is so high that it is critical for effective and rational use of ECMO by an experienced heart team.^12^ By contrast, after the successful removal of ECMO, more attention should be paid to postoperative management in order to improve postoperative outcomes, such as the use of medication for heart failure, comprehensive intervention for comorbidities, prolonged cardiac rehabilitation, and close outpatient follow-up. Our patient presented postoperative consciousness disorder, and CT showed no signs of cerebral infarction or cerebral hemorrhage, which may well be caused by an excessive use of sedatives. Therefore, we decided to discontinue sedatives and give neurotrophic therapy. Early transfer out of intensive care unit can reduce the incidence of cross-infection and iatrogenic infection after operation. In addition, early postoperative rehabilitation exercises, such as getting out of bed, lung vibration, and sputum drainage can also prevent pulmonary infection and lower limb venous thrombosis.

In conclusion, our experience can provide a solution for such patients. However, studies conducted with large samples are still needed to find the best treatment.

## Conclusion

TAVI assisted by ECMO may be a reasonable choice for patients with preoperative severe aortic stenosis complicated with low EF, heart failure, or even cardiogenic shock. Meanwhile, reasonable postoperative management can effectively prevent ECMO-related complications and improve the prognosis of patients.
